# Supplementary material for: Patient-Derived Bladder Cancer Organoid Models in Tumor Biology and Drug Testing: A Systematic Review
Source: Cancers (Basel). 2022 Apr 20;14(9):2062. doi: 10.3390/cancers14092062 (PMC9104249; doi:10.3390/cancers14092062)
Supplement: Supplementary file 1 [file cancers-14-02062-s001.zip › Supplemmentary Table S1.pdf]

Supplementary Material

Supplementary Table S1. Search queries and number of records retrieved from each database at 25/11/21.

Database	Search Query	Filter	Number of records
**PubMed**	((bladder cancer[MeSH Terms] OR urothelial OR urothelium) AND (organoids[MeSH Terms] OR spheroid))	("2000/01/01"[Date - Publication]: "2002"[Date - Publication])	101
**Embase**	('bladder cancer'/exp OR 'urothelial bladder cancer'/exp OR 'urothelial cancer'/exp OR 'bladder'/exp OR 'urothelium'/exp) AND ('organoid'/exp OR 'tumor spheroid'/exp OR 'spheroid':ti,ab,kw)	[2000-2021]/py AND ([article]/lim OR [article in press]/lim)	75
**Web of Science**	All fields((bladder OR urothelial OR urothelium) AND (organoid OR spheroid))	PUBYEAR > 1999<br>English, Articles only	182
**Scopus**	TITLE-ABS-KEY ((bladder OR urothelial OR urothelium) AND (organoid OR spheroid))	PUBYEAR > 1999<br>LIMIT-TO (DOCTYPE, "ar")<br>LIMIT-TO (LANGUAGE, "English")	176
**Unique entries**			246
